# Loss of Serum Glucocorticoid-Inducible Kinase 1 SGK1 Worsens Malabsorption and Diarrhea in Microvillus Inclusion Disease (MVID)

**DOI:** 10.3390/jcm11144179

**Published:** 2022-07-19

**Authors:** Md Kaimul Ahsan, Diego Carlos dos Reis, Andrea Barbieri, Kaelyn D. Sumigray, Timothy Nottoli, Pedro J. Salas, Nadia A. Ameen

**Affiliations:** 1 Department of Pediatrics, Gastroenterology and Hepatology, Yale University School of Medicine, New Haven, CT 06510, USA; mdkaimul.ahsan@yale.edu (M.K.A.); diegocarlos.dosreis@yale.edu (D.C.d.R.); 2 Department of Pathology, Yale University School of Medicine, New Haven, CT 06510, USA; andrea.barbieri@yale.edu; 3 Department of Genetics, Yale University School of Medicine, New Haven, CT 06510, USA; kaelyn.sumigray@yale.edu; 4 Genome Editing Center, Comparative Medicine, Yale University School of Medicine, New Haven, CT 06510, USA; timothy.nottoli@yale.edu; 5 Department of Cell Biology, Miller School of Medicine, University of Miami, Miami, FL 33146, USA; psalas@med.miami.edu; 6 Department of Cellular and Molecular Physiology, Yale University School of Medicine, New Haven, CT 06510, USA

**Keywords:** SGK1, MVID, CFTR, diarrhea

## Abstract

Microvillus inclusion disease (MVID), a lethal congenital diarrheal disease, results from loss of function mutations in the apical actin motor myosin VB (MYO5B). How loss of MYO5B leads to both malabsorption and fluid secretion is not well understood. Serum glucocorticoid-inducible kinase 1 (SGK1) regulates intestinal carbohydrate and ion transporters including cystic fibrosis transmembrane conductance regulator (CFTR). We hypothesized that loss of SGK1 could reduce CFTR fluid secretion and MVID diarrhea. Using CRISPR-Cas9 approaches, we generated R26^Cre^ER;MYO5B^f/f^ conditional single knockout (cMYO5BKO) and R26^Cre^ER;MYO5B^f/f^;SGK1^f/f^ double knockout (cSGK1/MYO5B-DKO) mice. Tamoxifen-treated cMYO5BKO mice resulted in characteristic features of human MVID including severe diarrhea, microvillus inclusions (MIs) in enterocytes, defective apical traffic, and depolarization of transporters. However, apical CFTR distribution was preserved in crypts and depolarized in villus enterocytes, and CFTR high expresser (CHE) cells were observed. cMYO5BKO mice displayed increased phosphorylation of SGK1, PDK1, and the PDK1 target PKCι in the intestine. Surprisingly, tamoxifen-treated cSGK1/MYO5B-DKO mice displayed more severe diarrhea than cMYO5BKO, with preservation of apical CFTR and CHE cells, greater fecal glucose and reduced SGLT1 and GLUT2 in the intestine. We conclude that loss of SGK1 worsens carbohydrate malabsorption and diarrhea in MVID.

## 1. Introduction

Microvillus inclusion disease (MVID) is a lethal inherited diarrheal disease of newborns resulting from loss of function mutations in the actin motor myosin VB (MYO5B) [1,2]. Affected infants develop uncontrolled diarrhea within hours of birth that resembles cholera and they die from massive fluid loss and dehydration unless they receive total parenteral nutrition (TPN) support for life. Intestinal fluid loss in MVID is both secretory and malabsorptive and often exceeds 140 mL/kg/day [3,4,5].

MYO5B is an unconventional actin motor myosin that associates with apical recycling endosomes (ARE) where it regulates trafficking of cargo to the apical plasma membrane of epithelial cells [6,7]. Apical trafficking defects in carbohydrate, ion, and other transporters were first identified in human MVID intestine eight years before MYO5B was identified [8]. Despite its importance to apical traffic in epithelial tissues including lung, liver, kidneys, loss of MYO5B leads to disproportionately overwhelming disease in the intestine.

In addition to its impact on apical traffic, loss of MYO5B leads to defects in important apical signaling pathways at the level of the ARE, including phosphoinositide kinase 1 (PDK1) and downstream targets [9]. Serum glucocorticoid kinase 1 (SGK1), a downstream target of PDK1, regulates proteins and apical transporters involved in intestinal absorption and secretion [10,11,12,13]. CFTR chloride channels on the apical membrane of intestinal cells are the major source of cyclic nucleotide (cAMP, cGMP)-activated Cl^−^ and H_3_CO^−^ secretion that underlies secretory diarrhea. MYO5B is necessary for CFTR traffic into the apical membrane of lung epithelial cells [7], and early studies in cultured intestinal epithelial cells suggested selectivity of MYO5B in regulating apical cargo and a requirement of MYO5B for CFTR traffic to the apical membrane [14]. However, studies from our laboratory instead demonstrated that in human MVID intestine CFTR traffics into the brush border membrane and is functional [15]. Similar observations were confirmed in an inducible MYO5BKO mouse model, a porcine model, and human MVID [5,16,17]. Differences therefore exist in MYO5B-dependent traffic of CFTR in lung vs. intestine that may account for lack of lung disease in MVID.

Available data from independent studies now firmly established that loss of MYO5B in enterocytes leads to malabsorption of ions, carbohydrates, and nutrients as well as active secretion of Cl^−^ and H_3_CO^−^ by CFTR. Because of its central role in secretory diarrheas [18], the combined effects of both malabsorption and CFTR-mediated secretion results in severe life-threatening diarrhea in MVID. But the relative contribution of CFTR-mediated fluid secretion or malabsorption to MVID diarrhea is unknown. In order to develop effective pharmacotherapy for MVID, it is critical to understand the relative contribution of fluid secretion vs. malabsorption.

SGK1 regulates intestinal Na^+^ coupled glucose transporter 1 (SGLT1) transport and the Na^+^/H^+^ exchanger 3 (NHE3), the major mechanism of electroneutral Na^+^ absorption in the small intestine [12]. Although loss of Na^+^ absorption due to defective apical traffic of NHE3 was suggested to underlie MVID diarrhea, loss of NHE3 on the enterocyte brush border alone does not lead to severe diarrhea resembling MVID [19,20]. We recently demonstrated profound effects of SGK1 activation on CFTR protein expression, brush border membrane traffic and function in the native intestine [21]. To understand the role of SGK1 in MVID diarrhea, the current study generated tamoxifen-inducible cMYO5BKO and cMYO5B-SGK1 double knockout mouse models (cSGK1/MYO5B-DKO) to examine the impact of loss of MYO5B alone compared with loss of both SGK1 and MYO5B on severity of diarrhea, carbohydrate malabsorption and ion transporters in the intestines.

## 2. Materials and Methods

### 2.1. Antibodies and Reagents

The protease inhibitors used for lysate preparation for the immunoblots, PhosSTOP EASYpack (#04906837001), and complete EDTA-free (#11873580001) were purchased from Roche. Dexamethasone (#1126) was purchased from Tocris Bioscience. Antibodies against phospho-NDRG1^Thr346^ (#5482), NDRG1 (#5196), phospho-PDK1^Thr346^ (#3061), PDK1 (#3062), and GAPDH (#8884) were purchased from Cell Signaling. Antibodies against Phospho-PI3K p85^Tyr458^/p55^Tyr199^ (#PA5-17387), GLUT2 (#PA5-77459), Phospho-SGK1^Thr256^ (#44-1260G), and Amplex™ Red Glucose Assay kit (#A22189) were purchased from Thermo Fisher Scientific, Waltham, MA, USA. Additional antibodies: CFTR (AME4991, affinity purified polyclonal antibody raised against rat CFTR-produced by Dr. Ameen), NHE3-3H3 (#MABN1813) and SGK1 (#07-315) from EMD Millipore (Burlington, MA, USA), Phospho-PKCι^Thr563^ (iota; #GTX25813) from GeneTex (San Antonio, TX, USA), Anti-SGLT1 (#ab14686) from Abcam (Cambridge, UK), and Myosin VB (#NBP1-87746) from Novus Biologicals (Centennial, CO, USA). Horseradish peroxidase-conjugated secondary antibodies were purchased from BD Bioscience (San Jose, CA, USA). Other reagents used in this study including tamoxifen (#T9262) were purchased from Sigma Aldrich (Burlington, MA, USA).

### 2.2. Generation of Tamoxifen-Inducible R26^Cre^ER;MYO5B^f/f^ Conditional Single Knockout (cMYO5BKO) and R26^Cre^ER;MYO5B^f/f^;SGK1^f/f^ Double Knockout (cSGK1/MYO5B-DKO)

**Figure 1:**
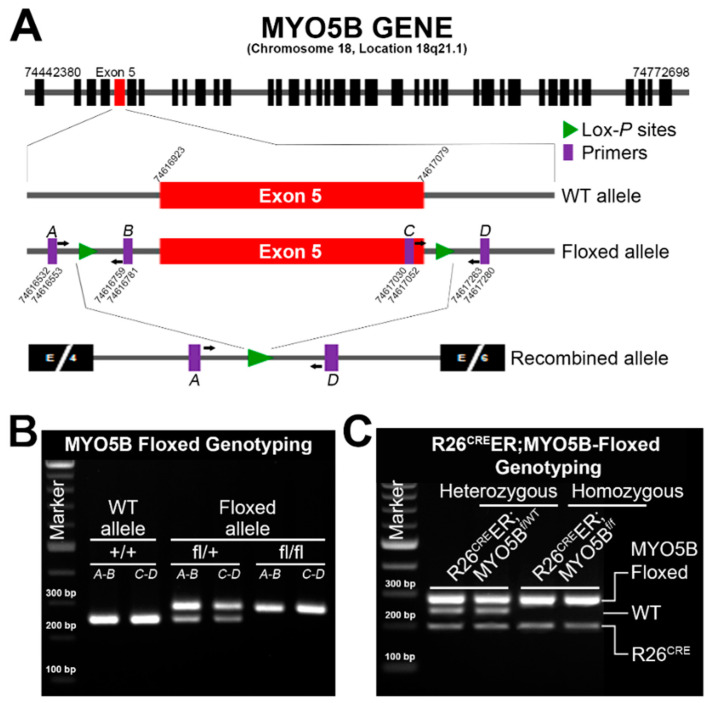
Generation of MYO5B^f/f^ (floxed) and inducible MYO5B conditional knockout (cMYO5BKO) mice. (**A**) Schematic diagram of the strategy for generating MYO5B^f/f^ and MYO5BKO mice by targeting and deleting Exon 5 using the CRISPR-Cas9 genome editing system. The purple-colored A–D primers are used to amplify the left lox-P and right lox-P sites, respectively for the genotyping protocol. Exons are indicated in black except for exon 5, which is shown in red. Lox-P sites are represented by green triangles. Combined allele was generated by crossbreeding MYO5B^f/f^ mice with R26^Cre^ER mice (B6;129-Gt(Rosa)26Sor^tm1(cre/ERT)Nat^/J), a tamoxifen-inducible Cre germline). (**B**) A representative agarose gel image of genomic DNA PCR shows MYO5B^f/f^ mice genotyping results with the different PCR product patterns. (**C**) A representative agarose gel image of Genomic DNA PCR shows R26^Cre^ER;MYO5B^f/f^ mice genotyping results with the different PCR product patterns used to identify heterozygous or homozygous mice.

Creation of the conditional MYO5B-floxed allele was performed via CRISPR/Cas9-mediated genome editing essentially as described [22]. To generate a floxed allele of exon 5 (Figure 1A), potential Cas9 target guide (protospacer) sequences in introns 4 and 5 were screened using the MIT CRISPR tool (http://crispr.mit.edu, accessed on 1 May 2021). The protospacer sequences GCACATAGTAGGCCTGTTGT (intron 4, forward strand) and GAAGACGGTCGACCTAACAC (intron 5, reverse strand) were chosen due to proximity to exon 5 and overall specificity scores. sgRNAs incorporating these protospacers were transcribed in vitro using the MEGAShortscript kit (Invitrogen, Waltham, MA, USA). Cas9 mRNA was similarly transcribed. Transcribed RNAs were purified using MEGAclear kit (Invitrogen) and eluted using microinjection buffer (10 mM Tris-HCl, pH 7.4; 10 mM NaCl and 0.25 mM EDTA, pH 8.0). The 154 bp repair template oligonucleotides (ssODN) containing loxP sites were synthesized by IDT Technologies. Components were mixed in injection buffer at a concentration of 30 ng/µL Cas9 mRNA, 15 ng/µL each sgRNA, and 30 ng/µL ssODN, centrifuged, and microinjected into the cytoplasm of C57BL/6J zygotes. Embryos were transferred to the oviducts of pseudopregnant CD-1 foster females using standard techniques [23]. Founder animals were identified by PCR and sequencing of the region spanning each loxP target site, and founders were mated to C57Bl/6J mice to confirm correct targeting and germline transmission of the conditional cKO allele.

For intestinal epithelium-specific MYO5B-KO mice, MYO5B^fl/fl^ mice were crossed with mice carrying the inducible R26-CreERT2 allele, which allows deletion of exon 5 in all epithelial cells of the intestine upon tamoxifen administration. Cre recombinase was activated by intraperitoneal injection of mice (16–20 weeks) with 2 mg tamoxifen per day for 3–5 days and weights were recorded daily just before tamoxifen injection using a digital balance machine. Mice were genotyped using genomic DNA PCR with specific primer designs enlisted in Table 1.

All animal procedures and study were approved by the Institutional Animal Care and Use Committee of Yale University School of Medicine.

### 2.3. Semiquantitative RT-PCR Analysis

To analyze MYO5B and SGK1 mRNA expression, total RNA was isolated using Trizol reagent (Invitrogen) from mucosal scrapings of mouse small intestines following treatment with tamoxifen (2 mg/mouse/day) for 3–5 days. About 3 µg of mRNA was used to synthesize cDNA using a SuperScript First-Strand Synthesis System (Invitrogen) with oligo dT12-18 according to the manufacturer’s protocol. The cDNA was amplified by normal PCR using a Taq-DNA polymerase (Qiagen) with a standard protocol where PCR band intensities were not saturated. PCR was performed using the following primers: MYO5B, 5′-ATGGAGCCGAACATCAATGCC-3′ (forward) and 5′-AGATGTTCCACCTCCTCCTCATG-3′ (reverse); SGK1, 5′-TGTGGCACGCCTGAGTATCTGGCT-3′ (forward) and 5′-CAGCCTCTTGGTCCGGTCCTTCTG-3′ (reverse);s GAPDH, 5′-GTATGACTCCACTCACGGCA-3′ (forward) and 5′-ATCACGCCACAGCTTTCCAGA-3′ (reverse); under following conditions: 28 cycles for MYO5B (denaturing at 94 °C for 30 s, annealing at 55 °C for 30 s, and extension at 72 °C for 40 s), 23 cycles for SGK1 (denaturing at 94 °C for 30 s, annealing at 60 °C for 30 s, and extension at 72 °C for 40 s), and 19 cycles for GAPDH (denaturing at 94 °C for 30 s, annealing at 58 °C for 30 s, and extension at 72 °C for 50 s). The PCR products were visualized by electrophoresis on 2% agarose gel (Sigma-Aldrich, Burlington, MA, USA) containing 1 µg/mL ethidium bromide.

### 2.4. Immunoblot Analysis

Mucosal scrapings from mouse small intestines were homogenized in TGH lysis buffer [25 mM HEPES, 10% (vol/vol) glycerol, 1% (vol/vol) Triton-X 100, pH 7.4)] containing a complete EDTA-free protease inhibitor cocktail (1×), 1 mM phenylmethylsulfonylfluoride (PMSF), plus Phos-STOP easy pack cocktail (1×) for 30 min on ice. Homogenates were solubilized, subject to centrifugation at 13,200 rpm for 15 min at 4 °C, and cleared supernatants were recovered. Protein concentration of the supernatant was determined with Coomassie Protein Assay Reagent (Pierce) and two sets (one set was warmed at 37 °C and used to detect CFTR and another set was boiled and used to detect kinases) of samples were prepared and analyzed as described [21].

### 2.5. Hematoxylin and Eosin (H&E) Staining

Jejunal tissues were removed from anesthetized animals. The intestinal lumens were washed with 1× PBS, cut into 1–1.5 cm in length, and tissues were fixed with 10% formalin immediately for 24 h and embedded in paraffin. Formalin-fixed paraffin-embedded tissues were sectioned at 4~5 μm of thickness and sections were stained with H & E and examined by light microscopy at ×40 magnification.

### 2.6. Transmission Electron Microscopy

Pieces of intestinal (jejunum) tissues were excised and prepared for transmission electron microscopy (TEM) by fixing in 2.5% glutaraldehyde in 0.1 mM cacodylate buffer at room temperature for 1 h followed by overnight fixation at 4 °C. Samples were washed and incubated with 1% osmium tetroxide (pH 6.0) for 1 h, stained with 1% uranyl acetate overnight at 4 °C followed by ethanol dehydration (30%, 50%, 70%, 95%, and 100%). Samples were further dehydrated in propylene oxide. After dehydration, tissue pieces were infiltrated with and embedded in EM bed 812 resin (cat no. 14120; Electron Microscopy Sciences, Hatfield, PA, USA). After solidification, blocks were sectioned on the ultramicrotome at 60–70 nm setting (silver or silver-gold colored section appearance) and grids with sections were stained with 2% uranyl acetate for 20 min and Reynold’s lead citrate for 1 min at room temperature. Grids were examined under the electron microscope equipped with Hamamatsu Orca HR camera and AMT camera system, HV 80.0 kV.

### 2.7. Immunohistochemistry

Formalin (10%)-fixed paraffin-embedded jejunal tissues from tamoxifen (2 mg/day/mouse)-treated C57BL/6J (WT), R26^Cre^ER;MYO5B^f/wt^ (Het), and R26^Cre^ER;MYO5B^f/f^ (Homo) mice were sectioned at 5~8 μm of thickness and sections were deparaffinized in xylene and rehydrated through ethanol to distilled water. Antigen retrieval was performed with citrate buffer (DAKO, Carpinteria, CA, USA) for 30 min. Endogenous peroxidase was blocked with 3% hydrogen peroxide for 5 min at room temperature. The slides were rinsed with Tween-20-Tris-buffered saline solution between each of the following steps. The sections were incubated with primary antibodies (SGLT1 and Phospho-SGK1^Thr256^) overnight at 4 °C. The antibodies were then detected by use of EnVision+ (#K4001, DAKO) solution. Diaminobenzidine was used to detect the antibody complex (#K3468, DAKO). Negative control sections were incubated with isotype-matched immunoglobulins. The slides were subsequently counterstained with hematoxylin, dehydrated, and cover slipped with resin mounting media. Immunolabeled sections were examined by light microscopy on an Olympus BX51 at ×40 magnification. Digital images were acquired with an Olympus cell Sens Standard imaging software.

### 2.8. Tissue Preparation and Immunofluorescence Labeling

Jejunal tissues from tamoxifen or vehicle-treated mice were removed from all groups, cut into 2-mm thick segments and fixed in 2% paraformaldehyde (PFA) in PBS for 30 minutes (min) at room temperature (RT-shaking), then changed to 0.2% PFA for 1h at RT, and rinsed three times with PBS. Intestinal segments were then cryoprotected in 20% sucrose overnight at 4 °C, shaking as previously described [24]. Tissue segments were embedded in cryomolds (#4566; Tissue-Tek, Torrance, CA, USA) containing optimal cutting temperature (OCT, #4583; Tissue-Tek, CA, USA) medium and then frozen by immersing molds in liquid nitrogen-cooled isopentane. Tissue blocks were stored at −20 °C and sectioned (5 µm) onto poly-lysine precoated Fisherbrand Superfrost®Plus slides (#12-550-15; Fisher Scientific) prior to immunolabeling. All immunolabeling steps were carried out in a humidified chamber at RT as previously described [24] except primary antibody incubation, which was carried out at 4 °C for overnight. Mounted cryosections (5 µm) were rehydrated with PBS and then treated with 1% Na borohydride for 10 min. Sections were washed with each solution: PBS, PBS/0.15% glycine (sol A), and PBS/1% BSA/0.15% glycine (sol B). Subsequent steps were carried out in sol B. To reduce non-specific binding, sections were blocked for 1 h at RT with normal goat serum (1:20) diluted in sol B and then washed. Cryosections were incubated with primary antibodies diluted in sol B + 0.2% Triton X-100 overnight at 4 °C. Negative control sections were labeled in the absence of primary antibodies. Sections were washed and then incubated for 1 h with Alexa Fluor-488 (green) conjugated secondary antibodies (mouse or rabbit) diluted 1:200 in sol B. F-actin staining was detected with Rhodamine Phalloidin. Tissue sections were washed with PBS, then cover slipped with ProLong™ Gold antifade mounting reagent with DAPI (#P36935, Invitrogen) and allowed to dry overnight at 4 °C in dark.

### 2.9. Fluorescence Microscopy

Immunolabeled sections were examined using a Zeiss AxioImager M2 microscope with Apotome.2 attachment, Axiocam 506 mono camera, P Apo 10X/0.45, Plan-Apochromat 20X/0.8, Plan-Apochromat 40X/1.3 Oil, Plan-Apochromat 63/1.4 Oil objectives and Zen software. Images were captured using Zeiss Zen 3.0 software. Scale bars were added based on objectives.

### 2.10. Glucose Measurement

Under anesthesia, the abdomen of tamoxifen/vehicle-treated animals was opened and whole intestines were removed in a Petri dish. Fecal luminal fluid was collected from each animal of every group, centrifuged at 13,200 rpm for 15 min at 4 °C and supernatant stored at −20 °C until assayed. Glucose concentrations were measured using an Amplex™ Red Glucose Assay Kit (#A22189; Thermo Scientific, Waltham, MA, USA) according to the manufacturer’s instructions. In brief, 50 µL of the reaction solution (10 mM Amplex Red, 10 U/mL HRP, 100 U/mL glucose oxidase, 50 mM sodium phosphate buffer, pH 7.4) was added to 50 µL of diluted fecal fluid in a 96-well microplate and incubated in the dark for 30 min at room temperature. The absorbance was then measured at 560 nm using a SpectraMax microplate reader (Molecular Devices, San Jose, CA, USA). Glucose concentration of luminal fecal fluid was determined from a standard curve generated using various concentrations (0–200 µM) of standard glucose solution.

### 2.11. Statistical Analysis

All data are shown as means ± SDs. Statistical significances were analyzed using Student’s *t*-test in the GraphPad Prism software (Version 7.0) and considered significant when the *p* value is <0.05.

## 3. Results

### 3.1. Generation and Characterization of a Conditional Tamoxifen-Inducible MYO5B Knock out Mouse Model of MVID

**Figure 2:**
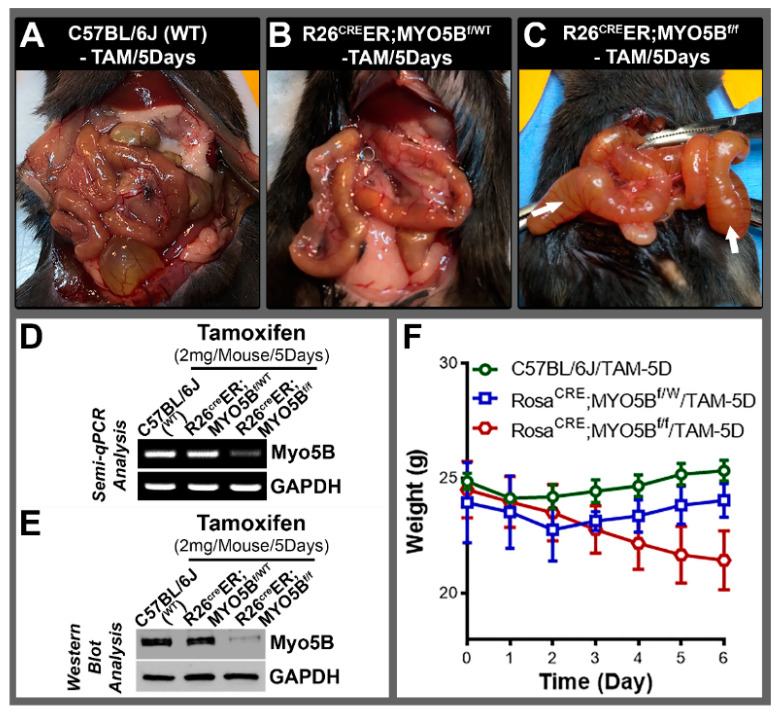
Tamoxifen induction at 5 days results in severe diarrhea in R26^Cre^ER;MYO5B^f/f^ (Homozygous) mice. A representative images of mouse intestines of (**A**) C57BL/6J (WT), (**B**) R2^Cre^ER;MYO5B^f^^/wt^ (Heterozygous), and (**C**) R26^Cre^ER;MYO5B^f/f^ (Homozygous) mice following 5 days treatment with tamoxifen (2 mg/day/mouse). Tamoxifen-treated homozygous mouse intestine display markedly distended loops with fluid-filled bowel (arrows) and severe diarrhea. (**D**) Semi-QRT-PCR analysis of mRNA from mucosal scrapings of tamoxifen-treated WT, R26^Cre^ER;MYO5B^f/wt^ and R26^Cre^ER;MYO5B^f/f^ jejunum confirmed loss of Myo5B mRNA expression in the R26^Cre^ER;MYO5B^f/f^ mice. GAPDH was used as a loading control. (**E**) Immunoblots of mucosal lysates from small intestines confirmed the loss of MYO5B protein in tamoxifen-treated R26^Cre^ER;MYO5B^f/f^ mice compared with WT and R26^Cre^ER;MYO5B^f/wt^ mice. GAPDH was used as a loading control. (**F**) Graph plots of animal weights over 6 days reveal significant weight loss in R26^Cre^ER;MYO5B^f/f^ mice. *N* = 6, independent experiments.

Several independent groups have generated and published data on mouse models of MVID. A global knockout mouse model of MYO5B [25] was first to demonstrate early lethality in mice due to severe diarrhea after birth. Subsequent models took advantage of inducible Cre recombinase to generate intestine-specific knockout models of MVID [5,17,26] by targeting exon 4 of MYO5B gene. Here, we developed an inducible mouse model of MVID by targeting exon 5 of MYO5B (Figure 1A). LoxP sites were introduced into intron 4 (forward strand) and intron 5 (reverse strand) via CRISPR-Cas9. Founder animals were identified by PCR (Figure 1B) and confirmed by sequencing, and the correct founders were mated to C57Bl/6J mice to confirm correct loxP targeting in cis by germline transmission. Next, the homozygous MYO5B^f/f^ (floxed) mice were crossbred with B6;129-Gt(ROSA)26Sor^tm1^^(Cre/ERT)Nat^/J (R26^Cre^ER) (Jackson, Stock No. 004847) germline and generated combined allele R26^Cre^ER;MYO5B^f/f^ (Figure 1C) to produce tamoxifen-inducible conditional MYO5B knockout (cMYO5B-KO) mice. Upon tamoxifen (2 mg/mouse/day) induction for 5 days, R26^Cre^ER;MYO5B^f/f^ mice displayed severe diarrhea with markedly distended fluid-filled intestine (Figure 2C) compared to heterozygous (Figure 2B) and WT (Figure 2A) mice. On day 4 of tamoxifen treatment, R26^Cre^ER;MYO5B^f/f^ mice had already began to appear ill and were lethargic. We collected intestinal mucosal scrapings to determine MYO5B expression level by semiquantitative QRT-PCR (Figure 2D) and immunoblot (Figure 2E). MYO5B expression was reduced by about 95% upon tamoxifen induction in the R26^Cre^ER;MYO5B^f/f^ (cMYO5BKO) (Figure 2D,E) mice compared to heterozygous and WT mice. Weight loss, an excellent measure of diarrhea, was significant in tamoxifen-induced R26^Cre^ER;MYO5B^f/f^ (Figure 2F) mice on day 5 compared to heterozygous and WT mice. Heterozygous and WT mice displayed slight reduction in weight initially, but that began to reverse on day 2/3 and gradually increased (Figure 2F).

**Figure 3:**
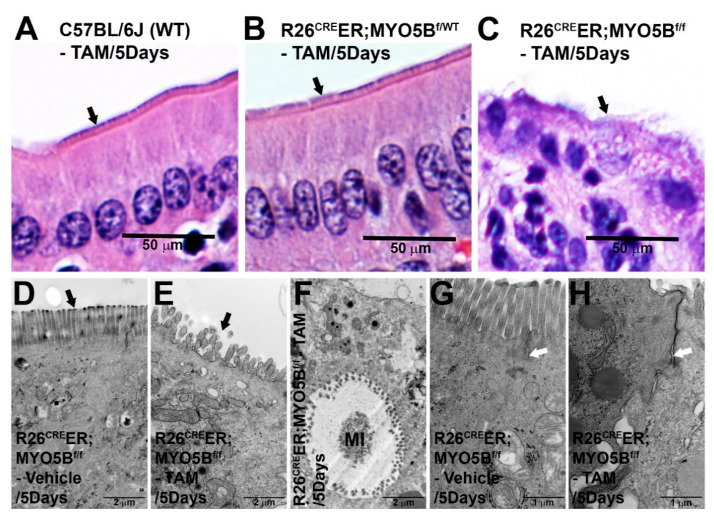
Histologic and ultrastructural changes in villus enterocytes of the small intestine induced by loss of MYO5B expression at day 5 of tamoxifen injection. H&E (**A**–**C**) staining of jejunum (**A**) C57BL/6J (WT), (**B**) R26^Cre^ER;MYO5B^f/wt^ (Heterozygous), and (**C**) R26^Cre^ER;MYO5B^f/f^ (Homozygous) mice treated with tamoxifen (2 mg/day/mouse) for 5 days. Shorter, aberrant microvilli (arrows) are observed in the tamoxifen-treated homozygous mouse jejunum compared to WT and heterozygous mice. Scale bars: 50 µm. (**D**–**H**) Transmission electron micrographs (TEM) show ultrastructure of microvilli, microvillus inclusion (MI), and tight-junction (TJ) in villus enterocytes from R26^cre^ER;MYO5B^f/f^ mice treated with vehicle (corn oil) or tamoxifen (2 mg/day/mouse) for 5 days. (**D**) Vehicle-treated R26^Cre^ER;MYO5B^f/f^ mouse jejunum display long, uniform microvilli on the apical surface of the enterocyte (arrow). Scale bar: 2 µm. (**E**) Tamoxifen-treated R26^Cre^ER;MYO5B^f/f^ mouse jejunum display shortened, disorganized, apical microvilli (arrow). Scale bar: 2 µm. (**F**) Microvillus inclusion (MI) typical of MVID is detected in the tamoxifen-treated R26^Cre^ER;MYO5B^f/f^ mouse jejunal villus enterocytes. Scale bar: 2 µm. (**G**) TJ (arrow) in vehicle-treated R26^Cre^ER;MYO5B^f/f^ mouse jejunal enterocytes. Scale bar: 1 µm. (**H**) TJ (arrow) in the tamoxifen-treated R26^Cre^ER;MYO5B^f/f^ mouse jejunal enterocytes. Image shows distended, widened inter-enterocyte spaces and prominent TJ compared to vehicle-treated R26^Cre^ER;MYO5B^f/f^ mouse jejunal enterocytes. Scale bar: 1 µm.

To characterize the efficacy of replicating features of human MVID in our cMYO5B KO mouse model, we used H&E staining and TEM analysis of small intestine to examine the characteristic morphologic and ultrastructural findings of human MVID. H&E staining of jejunum villus sections, shown in the upper panel (Figure 3A–C), revealed fully formed enterocyte brush borders (BB) in the 5-day tamoxifen-treated WT (Figure 3A) and heterozygous (Figure 3B) mice. In contrast, the R26^Cre^ER;MYO5B^f/f^ jejunum displayed immature appearing enterocytes with disorganized shorter brush borders (Figure 3C). TEM images of jejunal villus enterocytes from R26^Cre^ER;MYO5B^f/f^ (cMYO5BKO) (Figure 3E,F,H) shown in the lower panel, confirmed findings typical of human MVID. Microvilli on the apical surface of enterocytes were shorter, disorganized, and absent in some areas of the tamoxifen-treated R26^Cre^ER;MYO5B^f/f^ (cMYO5BKO) mouse model (Figure 3E), compared to the vehicle-treated R26^Cre^ER;MYO5B^f/f^ control mouse (Figure 3D). TEM analysis also identified several pathognomonic microvillus inclusions (MIs) in enterocytes from the 5-day tamoxifen-treated cMYO5BKO as shown in Figure 3F. TEM also revealed dilation of tight junctions (TJ) and widened inter-enterocyte villus spaces in the cMYO5BKO mouse model (Figure 3H) compared to the control mouse (Figure 3G).

### 3.2. Tamoxifen Induction in R26^Cre^ER;MYO5B^f/f^ (cMYO5BKO) Mice Downregulates SGLT1 Expression and Supports Carbohydrate Malabsorption

**Figure 4:**
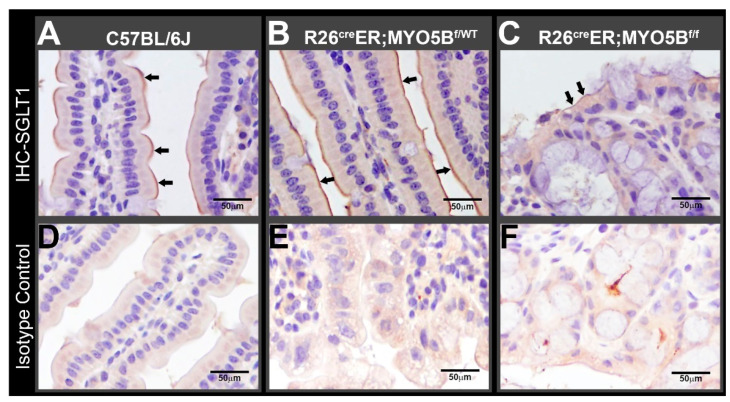
SGLT1 immunolabel is reduced in villus enterocytes from R26^cre^ER;MYO5B^f/f^ (Homozygous) mouse jejunum. Horseradish peroxidase staining detects SGLT1 (arrows) in jejunal villus sections from paraffin-embedded blocks of (**A**) C57BL/6J (WT), (**B**) R26^Cre^ER;MYO5B^f^^/wt^ (Heterozygous), and (**C**) R26^Cre^ER;MYO5B^f/f^ (Homozygous) mouse intestine. (**D**–**F**) are isotype controls for negative staining of (**A**–**C**), respectively. Arrows indicate SGLT1 labeling. Nuclei are stained blue. Scale bars: 50 µm.

The apical recycling endosome (ARE) is disrupted in human MVID due to defects in MYO5B. We demonstrated that disruption of the ARE leads to delocalization and activation of phosphoinositide-dependent kinase 1 (PDK1), atypical (aPKC) and downstream target serum glucocorticoid kinase 1 (SGK1) [27]. SGK1 regulates the sodium/glucose co-transporter 1 (SGLT1) [28] that mediates intestinal glucose absorption [29]. We examined the distribution of SGLT1 to determine whether the loss of MYO5B and disruption of ARE affect SGLT1 expression on the apical surface of enterocytes in our cMYO5BKO mouse model of MVID. Immuno-histochemistry labeling for SGLT1 revealed that it was distributed on the brush border of enterocytes in 5-day tamoxifen-treated WT (Figure 4A) and heterozygous (Figure 4B) mice, but was markedly reduced in the 5-day tamoxifen-treated R26^Cre^ER;MYO5B^f/f^ (cMYO5BKO) mice (Figure 4C), consistent with malabsorption of carbohydrates.

### 3.3. Tamoxifen Induction in R26^Cre^ER;MYO5B^f/f^ (cMYO5BKO) Mice Promotes Basolateral Redistribution of CFTR, Reduces Na^+^/H^+^ Exchanger 3 (NHE3) and Down-Regulated in Adenoma (DRA) Expression on the Villus Enterocyte Brush Border

**Figure 5:**
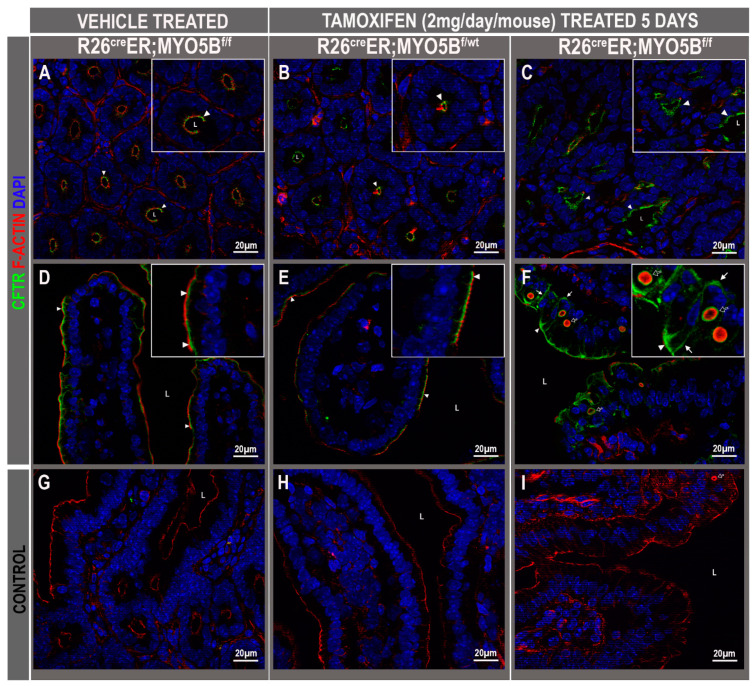
CFTR is preserved on the apical membrane and redistributed to the basolateral membranes of villus enterocytes in R26^Cre^ER;MYO5B^f/f^ (cMYO5bKO) jejunum. Immunofluorescence localization of CFTR and F-actin in immunolabeled cryostat sections from vehicle-treated R26^Cre^ER;MYO5B^f/f^, or tamoxifen (2 mg/day/mouse)-treated R26^Cre^ER;MYO5B^f/wt^ and R26^Cre^ER;MYO5B^f/f^ mice at day 5. Tissue sections from jejunum were immunolabeled for CFTR (green), F-actin (red), and DAPI (blue). (**A**,**D**,**G**) Images taken from vehicle-treated R26^Cre^ER;MYO5B^f/f^ (Homozygous) mice; (**B**,**E**,**H**) are from tamoxifen-treated R26^Cre^ER;MYO5B^f/wt^ (Heterozygous) mice; (**C**,**F**,**I**) are from tamoxifen-treated R26^Cre^ER;MYO5B^f/f^ (Homozygous) mice; (**G**–**I**) are negative control staining with secondary antibody only; (**A**–**C**) show CFTR staining in crypts, and (**D**–**F**) show CFTR staining in villus. The normal apical domain localization of CFTR (white arrowheads) in the villus of vehicle-treated homozygous (**D**) or tamoxifen-treated heterozygous jejunum (**E**), is redistributed to the basolateral membranes in villus enterocytes of tamoxifen-treated homozygous mouse jejunum (**F**, insert, white arrows). Several F-actin positive microvillus inclusions (MIs) (black arrows), decorated with CFTR on the outer membranes are detected in the cytoplasm of tamoxifen-treated R26^cre^ER;MYO5B^f/f^ mouse jejunal villus enterocytes. Inserts in upper right show higher power images of indicated areas in the lower power images. Scale bars: 20 µm.

To determine whether loss of MYO5B results in altered CFTR, NHE3, and DRA trafficking to the apical membrane of enterocytes similar to the observations in recent published animal models of MVID, immunolabel was performed in frozen jejunum sections of vehicle and tamoxifen-treated mice. CFTR was detected in the crypts and villus enterocytes of vehicle-treated R26^Cre^ER;MYO5B^f/f^ or, tamoxifen-treated R26^Cre^ER;MYO5B^f/wt^ (heterozygous) and R26^Cre^ER;MYO5B^f/f^ (cMYO5B KO) mice. Apical CFTR was preserved in the crypts (Figure 5A–C) and villi (Figure 5D–F) under all three conditions. However, CFTR was redistributed to the basolateral membranes of villus enterocytes in the R26^Cre^ER;MYO5B^f/f^ mice (Figure 5F). We observed several F-actin positive microvillus inclusions (MIs) clearly decorated with CFTR on the outer membranes in cMYO5BKO villus enterocytes (Figure 5F).

**Figure 6:**
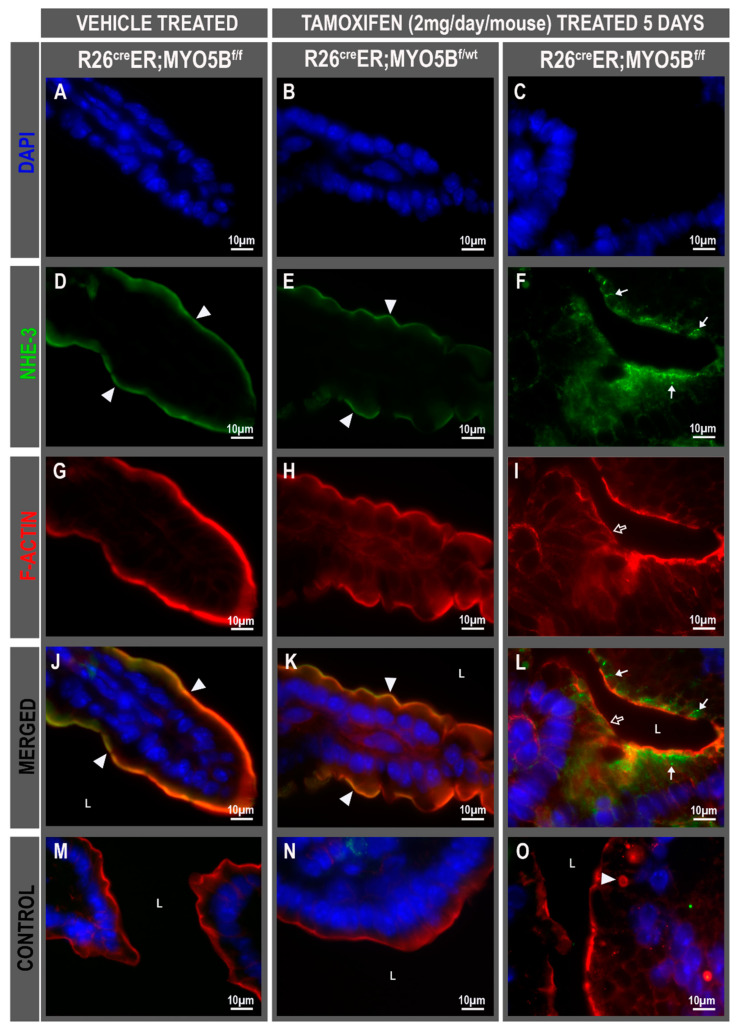
NHE3 is redistributed to subapical vesicular compartments in villus enterocytes of cMYO5bKO jejunum. Immunofluorescence localization of NHE3 in cryostat villus sections of jejunum from vehicle-treated R26^cre^ER;MYO5B^f/f^ or tamoxifen (2 mg/day/mouse)-treated R26^cre^ER;MYO5B^f/wt^ and R26^Cre^ER;MYO5B^f/f^ mice at day 5. Tissue sections were immunolabeled for NHE3 (green), F-actin (red), and DAPI (blue). Villus staining of DAPI (**A**–**C**) NHE3 (**D**–**F**) F-actin (**G**–**I**) merge (**J**–**L**) and control (**M**–**O**) from vehicle-treated R26^Cre^ER;MYO5B^f/f^ (Homozygous) (**A**,**D**,**G**,**J**,**M**) and tamoxifen-treated R26^Cre^ER;MYO5B^f/wt^ (Heterozygous) (**B**,**E**,**H**,**K**,**N**), or R26^Cre^ER;MYO5B^f/f^ (Homozygous) mouse jejunum (**C**,**F**,**I**,**L**,**O**). (**M**–**O**) Negative control staining with F-actin, Dapi, and secondary antibody only. The normal apical brush border localization of NHE3 (white arrowheads) observed in vehicle-treated homozygous (**D**), or tamoxifen-treated heterozygous mouse jejunum E, is redistributed to subapical vesicles (white arrows) in tamoxifen-treated homozygous mouse jejunum (**F**,**L**). (**O**) F-actin positive MI is detected (white arrowhead) in tamoxifen-treated homozygous mouse jejunum. Scale bars: 10 µm.

**Figure 7:**
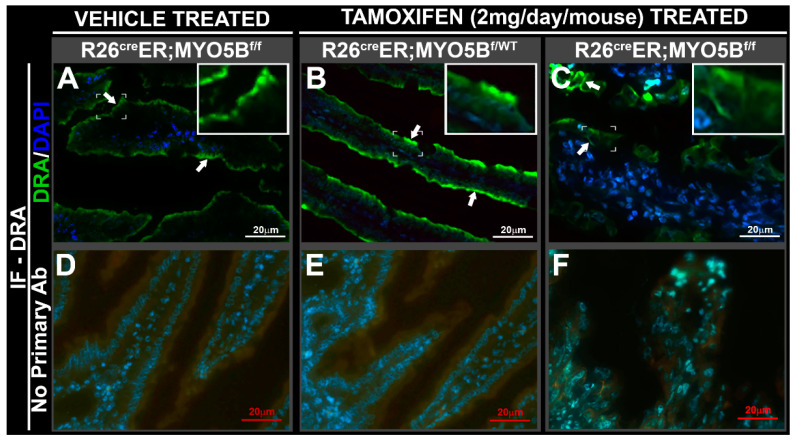
DRA is redistributed to the basolateral membranes in villus enterocytes of cMYO5BKO jejunum. Immunofluorescence localization of DRA in jejunum villus sections from vehicle-treated R26^Cre^ER;MYO5B^f/f^, or tamoxifen (2 mg/day/mouse)-treated R26^Cre^ER;MYO5B^f/wt^ and R26^Cre^ER;MYO5B^f/f^ mice at day 5. Cryostat jejunum sections were immunolabeled for DRA (green; arrows), and DAPI (blue). (**A**) Vehicle-treated R26^Cre^ER;MYO5B^f/^^f^ (Homozygous), and tamoxifen-treated (**B**) R26^Cre^ER;MYO5B^f/wt^ (Heterozygous), or (**C**) R26^Cre^ER;MYO5B^f/f^ (Homozygous) mouse jejunum. (**D**–**F**) Negative control staining of (**A**–**C**), respectively, stained with secondary antibody only. The normal apical brush border localization of DRA (arrows) observed in vehicle-treated homozygous, or tamoxifen-treated heterozygous mouse jejunum, is redistributed basolaterally in the tamoxifen-treated homozygous mouse jejunum. Brackets circumscribe the areas inserted under higher magnification. Scale bars: 20 µm.

The NHE3 (or SLC9A3) is the main regulator of Na^+^ absorption on villus enterocytes in the proximal small intestine, and like CFTR, is regulated by membrane traffic. Normal brush border localization of NHE3 was observed in the villus of vehicle-treated R26^Cre^ER;MYO5B^f/f^ (Figure 6D,J) and tamoxifen-treated R26^Cre^ER;MYO5B^f/wt^ (Heterozygous) (Figure 6E,K), but NHE3 brush border label was diminished and redistributed into cytoplasmatic vesicles of R26^Cre^ER;MYO5B^f/f^ (cMYO5BKO) mice (Figure 6F,L). Similar results were found for DRA (SLC26A3), the Cl^−^/HCO^3−^ exchanger responsible for Cl^−^ absorption and transport of SO_4_^2−^. R26^Cre^ER;MYO5B^f/f^ (cMYO5bKO) mice displayed DRA staining that was redistributed to the basolateral membranes of villus enterocytes (Figure 7C, and insert), while its apical localization was preserved on the villus in vehicle-treated R26^Cre^ER;MYO5B^f/f^ and tamoxifen-treated R26^Cre^ER;MYO5B^f/wt^ (Heterozygous) mice (Figure 7A,B).

### 3.4. Tamoxifen Induction in R26^Cre^ER;MYO5B^f/f^ (cMYO5BKO) Mice Increases SGK1 Phosphorylation in the Intestine

Disruption of the ARE, the site where MYO5B functions to regulate apical traffic delocalizes PDK1 and activates SGK1 [27]. SGK1 is also upregulated by non-genomic pathways under conditions of stress when cortisol levels are high and by phosphorylation through a phosphatidylinositol-3-kinase (PI3K)-phosphoinositide-dependent kinase 1 (PDK1) dependent mechanism [30,31,32]. SGK1 in turn mediates the stimulating effects on several transport systems by multiple mechanisms including kinase signaling, transcription, translation, membrane traffic, and membrane protein retention by ubiquitin ligases [33,34,35,36,37,38]. SGK1 exerts non-genomic regulation of absorptive transporters including SGLT1 and NHE3, but it also exerts a profound non-genomic effect on CFTR traffic, expression, and ion transport in the native small intestine [21].

**Figure 8:**
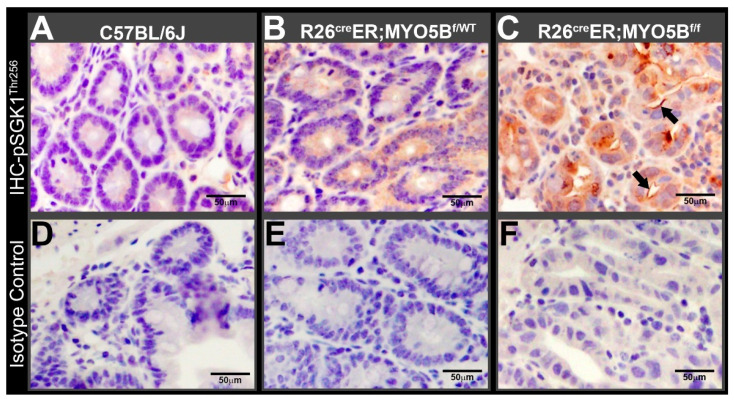
Phospho-SGK1^Thr256^ is increased in tamoxifen (2 mg/day/mouse for 5 days)-treated R26^Cre^ER;MYO5B^f/f^ (Homozygous) mouse jejunum. Horseradish peroxidase staining detects pSGK1^Thr256^ (arrows) in jejunal sections from paraffin-embedded blocks of (**A**) C57BL/6J (WT), (**B**) R26^Cre^ER;MYO5B^f/wt^ (Heterozygous), and (**C**) R26^Cre^ER;MYO5B^f/f^ (Homozygous) mice. (**D**–**F**) are isotype controls for negative staining of (**A**–**C**), respectively. Arrows are indicating pSGK1^Thr256^ labeling. Nuclei are stained blue. Scale bars: 50 µm.

Since our cMYO5BKO mouse model replicates human MVID and a role for SGK1 in MVID diarrhea has not been considered before, we used immunohistochemistry to examine the phosphorylation status of SGK1 in the small intestine of R26^Cre^ER;MYO5B^f/f^ (cMYO5BKO), compared to heterozygous and wild type mice. Using a phospho-specific antibody that detects phosphorylated SGK1^Ser256^, the specific site phosphorylated by PDK1 [34,35], we found marked increased phosphorylation of SGK1 on the apical domain of crypt epithelial cells in MYO5BKO mouse jejunum (Figure 8C) compared to heterozygous (Figure 8B) or wild type (Figure 8A). Isotype control staining, shown in lower panels (Figure 8D–F) were negative.

### 3.5. Immunoblot Changes in Protein Expression of Ion Transporters and Signaling Kinases in the Small Intestine of R26^Cre^ER;MYO5B^f/f^ (cMYO5B KO) Mice

**Figure 9:**
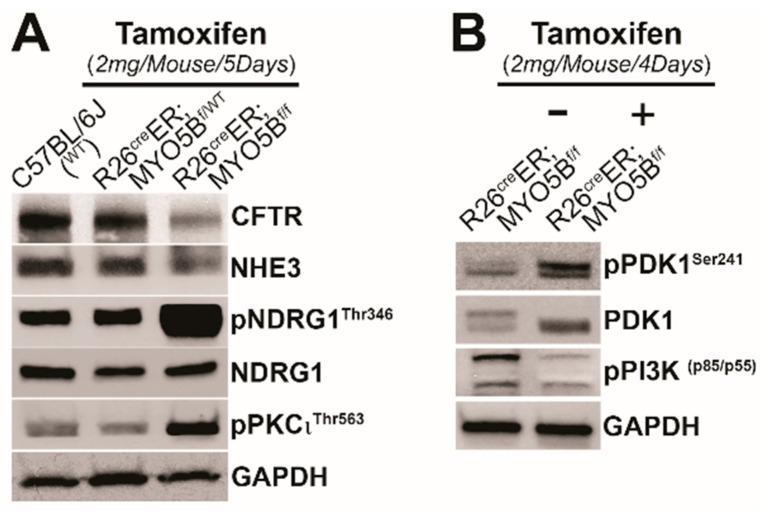
Immunoblots of ion transporters and phosphorylated kinases in C57BL/6J (WT), R26^Cre^ER;MYO5B^f/wt^, or R26^Cre^ER;MYO5B^f/f^ mice treated with tamoxifen (2 mg/day/mouse) for 4–5 days. (**A**) Immunoblots of CFTR, NHE3, NDRG1, pNDRG1^Thr346^, and pPKCι^Thr563^ in tamoxifen (2 mg/mouse/day)-treated C57BL/6J (WT), R26^Cre^ER;MYO5B^f/wt^ (Heterozygous) or R26^Cre^ER;MYO5B^f/f^ (Homozygous) mouse jejunal lysates at day 5. (**B**) Immunoblots of PDK1, pPDK1^Ser241^ and pPI3K p85^Tyr458^/p55^Tyr199^ in jejunal lysates of R26^Cre^ER;MYO5B^f/f^ mice treated with or without tamoxifen (2 mg/mouse/day) for 4 day, vs. vehicle (corn oil). GAPDH was used as a loading control for both experiments. *N* = 3, independent experiments for each condition examined.

To understand how changes in ion transporter protein expression and PDK1 associated kinase signaling pathways are linked to MVID diarrhea, we analyzed immunoblots of small intestine tissue lysates from wild type, heterozygous or R26^Cre^ER;MYO5B^f/f^ (cMYO5BKO) mice following tamoxifen treatment for 4 or 5 days. Total CFTR, and NHE3 protein were slightly reduced in tamoxifen-treated R26^Cre^ER;MYO5B^f/f^ (cMYO5BKO) mice at day 5 compared with WT or heterozygous mice. Consistent with the IHC observation (Figure 8) that phosphorylated SGK1 was increased in cMYO5BKO intestine, immunoblot of NDRG1 phosphorylation at Thr^346^, the specific site that is phosphorylated by SGK1, was markedly increased in lysates at day 5 in tamoxifen-treated R26^Cre^ER;MYO5B^f/f^ (cMYO5BKO) mice. Tissues from R26^Cre^ER, MYO5B^f/f^ cMYO5BKO also showed increased phosphorylation of PKCι^Thr563^ and PDK1^Ser241^ while phosphorylation of PI3K p85^Tyr458^/p55^Tyr199^ was reduced (Figure 9).

### 3.6. Conditional MYO5B-SGK1-DKO (cSGK1/MYO5B-DKO) Mice Display More Severe Diarrhea Compared with cMYO5BKO Mice

**Figure 10:**
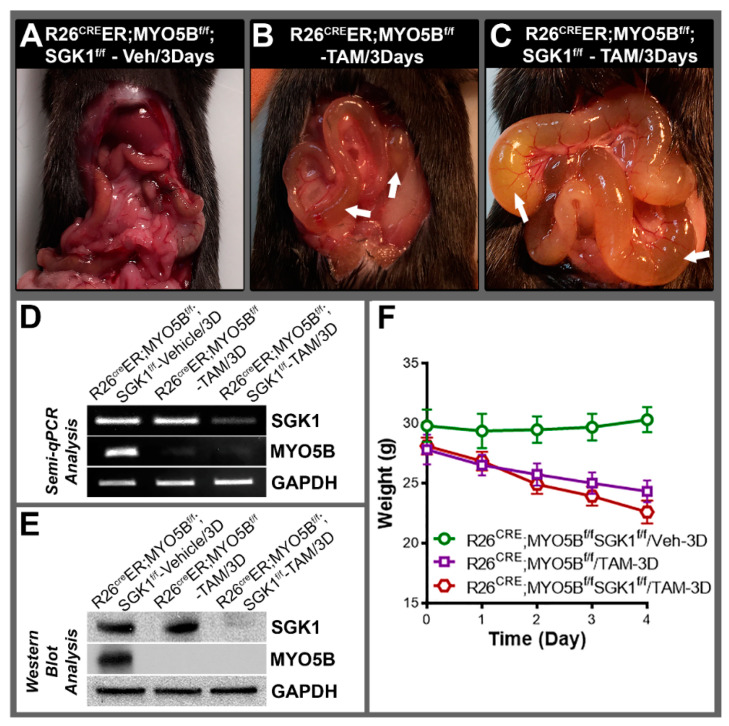
cMYO5B-SGK1 DKO mice display more severe diarrhea vs. cMYO5B KO at Day 3 of tamoxifen. Representative images of mouse intestines from (**A**) vehicle-treated R26^Cre^ER;MYO5B^f/f^;SGK1^f/f^, (**B**) tamoxifen-treated R26^Cre^ER;MYO5B^f/f^ (cMYO5BKO), and (**C**) tamoxifen-treated R26^Cre^ER;MYO5B^f/f^;SGK1^f/f^ (cMYO5B-SGK1DKO) mice following 3 days (2 mg/day/mouse) vs. treatment with vehicle (Corn oil). Intestines from tamoxifen-treated R26^Cre^ER;MYO5B^f/f^;SGK1^f/f^ (**C**) mice displayed the greatest distended loops with fluid-filled bowel (arrow) and increased diarrhea compared to R26^Cre^ER;MYO5B^f/f^ (**B**) mice and vehicle-treated R26^Cre^ER;MYO5B^f/f^;SGK1^f/f^ (**A**) mice that did not display diarrhea. (**D**) Semi-QRT-PCR analysis of mRNA from mucosal scrapings of vehicle or tamoxifen-treated R26^Cre^ER;MYO5B^f/f^;SGK1^f/f^, or R26^Cre^ER;MYO5B^f/f^ jejunum confirmed loss of mRNA expression of MYO5B in the tamoxifen-treated R26^Cre^ER;MYO5B^f/f^ mice and both MYO5B and SGK1 in the R26^Cre^ER;MYO5B^f/f^;SGK1^f/f^ mice compared to vehicle-treated R26^Cre^ER;MYO5B^f/f^;SGK1^f/f^ mice. GAPDH was used as a loading control. (**E**) Immunoblots of mucosal lysates from small intestines confirmed loss of Myo5B expression in the tamoxifen-treated R26^Cre^ER;MYO5B^f/f^ mice and loss of both MYO5B and SGK1 expression in the tamoxifen-treated R26^Cre^ER;MYO5B^f/f^;SGK1^f/f^ mice compared to vehicle-treated R26^Cre^ER;MYO5B^f/f^;SGK1^f/f^ mice. GAPDH was used as a loading control. (**F**) Graph plots show weights over 4 days in tamoxifen-treated R26^Cre^ER;MYO5B^f/f^ and R26^Cre^ER;MYO5B^f/f^;SGK1^f/f^ mice compared to R26^Cre^ER;MYO5B^f/f^;SGK1^f/f^ and R26^Cre^ER;MYO5B^f/f^ mice. *N* = 6, independent experiments.

We hypothesized that loss of SGK1 could reduce MVID diarrhea since SGK1 activation up-regulates CFTR. To test our hypothesis, we generated double conditional MYO5B-SGK1-DKO mice. Tamoxifen treatment of R26^Cre^ER;MYO5B^f/f^ (cMYO5BKO) mice for 5 days resulted in distended intestinal loops of bowel and severe diarrhea in mice as shown in Figure 2. Surprisingly, attempts at induction with tamoxifen treatment for 5 days in cMYO5B-SGK1-DKO mice proved unsuccessful, as mice became severely ill by day 3–4 and did not survive due to severe diarrhea, weight loss, and lethargy. We therefore compared diarrhea in cMYO5B vs. cMYO5B-SGK1-DKO following tamoxifen induction for 3 days (Figure 10A–C). Diarrhea and bowel distension from fluid accumulation was greater in cMYO5B-SGK1-DKO compared with cMYO5BKO, as reflected in greater weight loss in cMYO5B-SGK1-DKO vs. cMYO5BKO mice (Figure 10F). Loss of both SGK1 and MYO5B was confirmed in tissues from cSGK1/MYO5B-DKO mice by PCR and immunoblot analysis of small intestine lysates as shown in Figure 10D,E.

### 3.7. CFTR High Expresser (CHE) Cells Are Present in cMYO5BKO and cMYO5B-SGK1-DKO Mice

**Figure 11:**
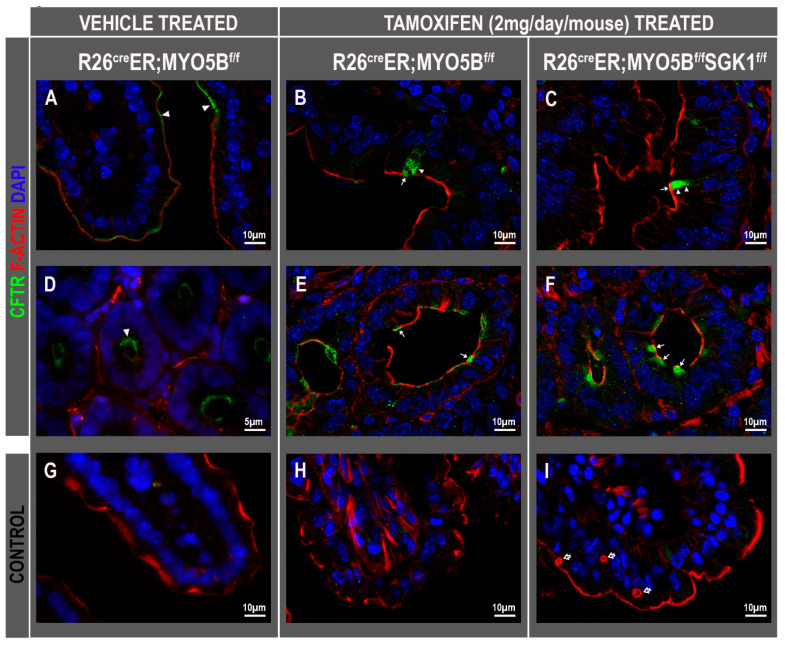
CFTR high expressor (CHE) cells are present in the jejunum of tamoxifen (2 mg/day/mouse for 3 days)-treated R26^Cre^ER;MYO5B^f/f^ (cMYO5BKO) and R26^Cre^ER;MYO5B^f/f^;SGK1^f/f^ (cMYO5B-SGK1-DKO) mice. Tissue sections were immunolabeled for CFTR (green), F-actin (red), and DAPI (blue). (**A**,**D**) Vehicle-treated R26^Cre^ER;MYO5B^f/f^, and tamoxifen-treated (**B**,**E**) R26^Cre^ER;MYO5B^f/f^ (cMYO5BKO) or, tamoxifen-treated (**C**,**F**) R26^Cre^ER;MYO5B;SGK1^f/f^ (cMYO5B-SGK1DKO) mice (**G**–**I**) are negative control staining with secondary antibody, F-actin, and DAPI. Apical and subapical localization of CFTR was found in the villus (**A**) and crypts (**D**) of vehicle-treated homozygous (arrowheads) and in CHE cells (arrows) of Tamoxifen-treated R26^Cre^ER;MYO5B^f/f^ (cMYO5BKO) (**B**,**E**) or R26^Cre^ER;MYO5B;SGK1^f/f^ (cMYO5B-SGK1-DKO) (**C**,**F**). Arrowheads in (**B**,**C**) show normal subcellular localization of CFTR in CHE cells. Multiple F-actin positive microvillus inclusions (MIs) typical of MVID are shown in villus enterocytes of R26^Cre^ER;MYO5B^f/f^ SGK1^f/f^ (cMYO5BKO) jejunum (**I**, empty arrow). Scale bars: 5 µm and 10 µm.

CHE cells were first reported in localization studies of CFTR in the human and rat intestine in 1995 [39] but were not found in mice under homeostatic conditions [40]. CHEs are unique rare enterocytes (~1–2% total epithelial cells) found both in crypts and villi but more predominantly found scattered along the villi of duodenum and jejunum where they express unusually high levels of CFTR both in subapical endosomes and on the brush border [39,41,42,43,44]. Unlike villus enterocytes of the small intestine, CHEs lack the typical absorptive markers that are present on the brush border, including sucrase isomaltase, lactase, alkaline phosphatase, but they express high levels of the vATPase (vacuolar H^+^-ATPase proton pump) and CFTR. Interestingly, villus CHEs display robust trafficking of endosomal associated CFTR into the brush border upon cAMP, cGMP, Ca^2+^ stimulation, and low pH consistent with a secretory profile [41,42,43,44]. We observed high levels of CFTR on the brush border of villus CHE cells in human MVID duodenal tissues resulting from loss of MYO5B mutations [15]. Consistent with our observations in human MVID, here we observed CHE cells with a normal subcellular distribution of CFTR on the villi (Figure 11B,C) and in superficial crypts (Figure 11E,F) of cMYO5BKO (Figure 11B,E) and also in the cMYO5B-SGK1-DKO (Figure 11C,F) mouse jejunum.

### 3.8. Loss of SGK1 Leads to Further Reduction of Glucose Transporter Expression and Increased Intestinal Fluid Loss in MVID

**Figure 12:**
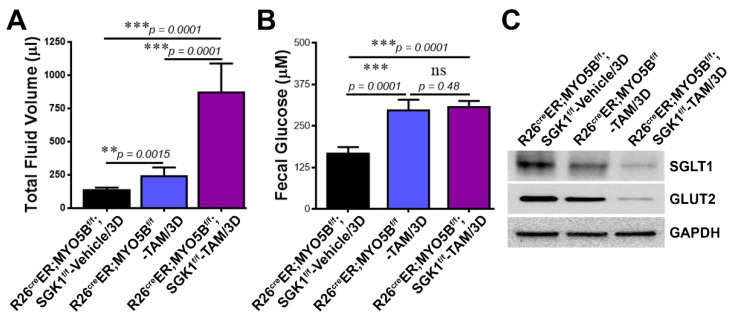
Increased diarrhea in cMYO5B-SGK1 DKO mice is associated with increased carbohy-drate loss. Fecal fluid volumes, fecal glucose and mucosal glucose transporter protein expression in tamoxifen (2 mg/day/mouse for 3 days)-induced R26^Cre^ER;MYO5B^f/f^ (cMYO5B-KO) and R26^Cre^ER;MYO5B^f/f^;SGK1^f/f^ (cMYO5B-SGK1-DKO) mice compared to vehicle-treated cMYO5B-SGK1-DKO mice. (**A**) Graphs of volumes of total fecal fluid from mice at day 3 of tamoxifen vs. vehicle induction. Fecal fluid volume was highest in tamoxifen-treated R26^Cre^ER;MYO5B^f/f^;SGK1^f/f^ (cMYO5B-SGK1-DKO) mice compared to R26^Cre^ER;MYO5B^f/f^ (cMYO5BKO) or vehicle-treated R26^Cre^ER;MYO5B^f/f^;SGK1^f/f^ mice. (**B**) Fecal fluid glucose levels were significantly higher in the 3 days tamoxifen-treated R26^Cre^ER;MYO5B^f/f^ (cMYO5BKO) and R26^Cre^ER;MYO5B^f/f^;SGK1^f/f^(MYO5B-SGK1-DKO) mice compared to vehicle-treated R26^Cre^ER;MYO5B^f/f^;SGK1^f/f^ mice. (**C**) Representative immunoblots of glucose transporters SGLT1 and GLUT2 confirm reduced protein ex-pression in intestinal mucosa in the tamoxifen-treated R26^Cre^ER;MYO5B^f/f^ (cMYO5BKO) intestine compared to vehicle-treated that is further reduced in R26^Cre^ER;MYO5B^f/f^;SGK1^f/f^ (cMYO5B-SGK1-DKO) mice. GAPDH was used as a loading control. *N* = 6, mice from each group.

MVID diarrhea is severe with high stool volume (150–300 mL/kg/day). The diarrhea is considered to be primarily non-osmotic (i.e., fecal ion gap < 100 mOsm) with elevated stool electrolytes consistent with electrolyte transport diarrhea [45], although malabsorption of carbohydrates is also observed due to reduced SGLT1 on the enterocyte brush border [5,16]. SGLT1 is the main mediator of intestinal glucose absorption on the brush border while GLUT2 facilitates basolateral exit [46]. SGK1 regulates CFTR, NHE3, and SGLT1 in the intestine [10,21,33]. To understand the contribution of SGK1 to carbohydrate loss in MVID diarrhea, we examined fecal fluid volumes, fecal glucose concentration, and protein expression of SGLT1 and GLUT2 in small intestinal mucosal lysates from heterozygous, tamoxifen-treated cMYO5BKO or cSGK1-MYO5B DKO mice. Fecal volume (Figure 12A) was highest in the cSGK1-MYO5B DKO mice, while fecal glucose was not markedly different between cMYO5BKO and cSGK1-MYO5B DKO. But expression of SGLT1 and GLUT2 in mucosal lysates from cSGK1-MYO5B DKO was markedly reduced compared with cMYO5BKO mice (Figure 12C).

## 4. Discussion

Since the MYO5B gene was discovered and implicated in MVID, several independent laboratories have successfully generated animal models of MVID. Several models confirmed that loss of MYO5B results in apical trafficking defects of ion and glucose transporters in small intestinal enterocytes similar to the findings in human MVID. The current study successfully used CRISPR/Cas9 approaches and deletion of Exon 5 in the MYO5B gene to generate an inducible mouse model of MVID that recapitulates intestinal disease in human MVID. Deletion of Exon 5 in MYO5B and use of ROSA*CreERT* resulted in a mouse model with many features resembling the first published VilCre tamoxifen-inducible mouse model resulting from deletion of Exon 4 of MYO5B [17].

Characteristic features of human MVID were observed in our cMYO5BKO mice including weight loss, severe diarrhea, brush border defects in villus enterocytes of the small intestine, pathognomonic microvillus inclusions (MIs), widened spaces between enterocytes, apical trafficking defects of ion transporters including DRA, NHE3, and reduced expression of the glucose transporter SGLT1. Similar to our previous observations in human MVID small intestine [15], we observed that apical CFTR was preserved in the crypts of cMYO5BKO mice. CFTR was also preserved on the brush border of cMYO5BKO villus enterocytes, but some of it was redistributed to the basolateral membranes consistent with MYO5B-dependent polarity defects. MYO5B is an actin motor myosin that functions at the apical recycling endosome (ARE) where it traffics apical cargo into the brush border. CFTR is an apical membrane protein that traffics into the apical brush border from the ARE. We previously showed that loss of MYO5B disrupts the ARE, and thus traffic of many apical directed proteins, including CFTR [9,15]. Consistent with our early observations in intestinal tissues of patients with MVID, where we observed CFTR in MIs [8], in the current study, cMYO5BKO mouse villus enterocytes clearly displayed CFTR decorating the MI membranes. MIs in MVID villus enterocytes display fully formed brush borders. The underlying pathogenesis of MI formation in MVID remains unresolved, but is suggested to be linked to differentiation [47] although MI’s are also observed in the less differentiated crypt, although less frequently compared to villus enterocytes [8].

An interesting and unique finding in our cMYO5BKO mouse model was the identification of CFTR high expresser cells (CHE) that we previously reported in normal [39] and MVID human small intestine [15]. Several CHEs were also identified in the crypt and villi of cSGK1-MYO5B-DKO mouse. The distribution of CFTR in CHEs in the crypt and villi of the cMYO5BKO and cSGK1-MYO5B-DKO mouse resembled its normal subcellular distribution, with abundant apical and subapical CFTR. Although several MYO5BKO mouse models have been generated by independent groups [5,17,26] as well as a pig model [16], this is the first documentation of the presence of CHEs in pathogenic mouse models. Why CHEs are present in pathogenic mouse models of MVID but not under homeostatic conditions is intriguing and will require further investigations. 

Why CFTR traffics to the brush border, is present in Mis, and retains its apical distribution in CHE cells in the absence of MYO5B are also unknown. Our localization studies of CFTR led to the first recognition of CHE cells in rat and human intestine, as they are not easily distinguished morphologically by light microscopy, or H&E staining. However, recent advances in scRNA-seq have facilitated recognition of CHEs and identified a number of genes responsible for ion transport and pH regulation including Guca2b and Best4 that are specific to CHEs in the human small intestine [48]. These observations suggest an important role for CHEs in contributing to fluid secretion in MVID.

The observed increase in phosphorylation of SGK1, PKCι, and PKD1 in cMYO5BKO intestine is consistent with previous observations that loss of MYO5B disrupts the ARE and apical PDK1 signaling pathway [9]. The explanation for the observed reduced activation of PI3K in the cMYO5BKO small intestine is unclear but could be linked to its role in traffic [21,49].

MVID diarrhea is severe, non-osmotic, and categorized as electrolyte transport defect [50], although malabsorption of ions and glucose contributes to its severity. SGK1 regulates absorptive apical proteins and ion transporters in the intestine including NHE3 and GLUT2, but it markedly upregulates CFTR expression, traffic, and ion transport [21]. We hypothesized that silencing SGK1 could potentially reduce the “secretory” component of MVID diarrhea. We were surprised that the diarrhea in cSGK1-MYO5B-DKO mice was more severe than that observed in cMYO5BKO mice. Loss of SGK1 resulted in further increased fecal loss and reduction of glucose malabsorption as evidenced by much lower SGLT1 and GLUT2 protein expression in cSGK1-MYO5B-DKO compared to cMYO5BKO tissues. Although fecal glucose levels were statistically unchanged in cMYO5B and cSGK1-MYO5B-DKO, this is likely a dilutional effect due to the high fecal fluid volume in the DKO. Nevertheless, the presence of CHEs in the crypt and villi in cSGK1-MYO5B-DKO mice was surprising and presents more challenge in dissecting the contributions of secretory and malabsorption processes in MVID as increased numbers of functional CHEs could also potentially contribute to the worsening of diarrhea. Further studies are required to understand CFTR brush border traffic in MVID, and the role of CHEs in MVID diarrhea.

## Figures and Tables

**Table 1:** Primers used in genotyping protocols.

Recombination Template Sequences
MYO5B 5′loxP template: (forward strand) 5′GAAGACCCTTGTTCTTATCAGAAAACTTATAGGGAATTCAGTGGCACATAGTAGGCCTGTATAACTTCGTATAATGTATGCTATACGAAGTTATTGTAGGAACGAAGACAAAAGGATAGATGTTCTTAGGACAATGGGCATCATGTCCCAGTTT 3′
MYO5B 3′loxP template: (reverse strand) 5′CCATCACTACAGCACGCACAGGGTCCAAGGACACAGAGAGCAT**GAAGACGGTCGACCTAA**ATAACTTCGTATAGCATACATTATACGAAGTTAT**CAC****AGG**GCCCTGCAGGACGATTATCTTTAACCCTGGGTTTCAGAGGTACCTCCACCCTCT 3′

## Data Availability

Not applicable.
